# Función Motora Gruesa Tras Rehabilitación Con Exoesqueleto Pediátrico Atlas 2030 En Niños Con Parálisis Cerebral

**DOI:** 10.31083/RN46141

**Published:** 2025-08-29

**Authors:** Irma García Oliveros, Nerea Meabe Iturbe, Juan Ignacio Marín Ojea, Carolina Lancho Poblador, Paola Fuentes-Claramonte, José Ignacio Quemada Ubis

**Affiliations:** ^1^Servicio de Daño Cerebral, Hospital Aita Menni, 48010 Bilbao, Bizkaia, Spain; ^2^Servicio de Daño Cerebral, Hospital Aita Menni, 20509 Mondragón, Gipuzkoa, Spain; ^3^Servicio médico, Centro Goienetxe, 20018 Donostia, Gipuzkoa, Spain; ^4^FIDMAG, Hermanas Hospitalarias Research Foundation, CIBERSAM ISCIII, 08830 Sant Boi de Llobregat, Spain

**Keywords:** exoesqueleto, rehabilitación, parálisis cerebral, pediatría, función motora gruesa, exoskeleton, rehabilitation, cerebral palsy, pediatrics, gross motor function

## Abstract

**Introducción y Objetivos:**

Evaluar el impacto del entrenamiento intensivo de marcha con el exoesqueleto pediátrico ATLAS 2030 en la función motora gruesa, así como determinar el mantenimiento de los efectos post-intervención en niños con parálisis cerebral (PC).

**Sujetos y Métodos:**

Estudio prospectivo controlado no aleatorizado. Participaron 13 niños con PC. Se implementó un programa de 4 sesiones semanales de 65 minutos durante 6 semanas. Se evaluó la función motora gruesa con la Gross Motor Function Measure de 88 ítems (GMFM-88), la resistencia física al ejercicio con el test de los seis minutos marcha (6MWT) con el dispositivo y se registró el número de pasos caminado en cada sesión en cada modo de uso para evaluar la adaptación a la actividad. Se realizaron 3 evaluaciones, antes del tratamiento, al finalizarlo (6 semanas) y una última evaluación de seguimiento a las 12 semanas.

**Resultados:**

La puntuación total de la GMFM-88 mostró cambios significativos al finalizar la intervención (*p* < 0,001), persistiendo en el seguimiento (*p* < 0,001). El número de pasos en automático y en activo aumentaron significativamente tras la intervención (*p* < 0,001), manteniéndose en el seguimiento (*p* = 0,001). Por último, el 6MWT aumentó de manera significativa tras la intervención, reduciéndose en el seguimiento (*p* < 0,001).

**Conclusiones:**

El entrenamiento intensivo de 6 semanas con ATLAS 2030 impacta positivamente en la función motora gruesa de niños con PC, aumentando los beneficios 6 semanas después de la finalización del tratamiento. La resistencia física y la adaptación a la actividad mejoran con el uso continuado. Estos resultados respaldan el potencial del ATLAS 2030 como estrategia terapéutica intensiva en esta población.

**Registro de Ensayos Clínicos:**

No: NCT07066956. https://clinicaltrials.gov/search?cond=NCT07066956.

## Abstract (en)

**Introduction and Objectives:**

To evaluate the impact of intensive gait training on gross motor function using the pediatric exoskeleton ATLAS 2030, as well as to determine the post-intervention maintenance of effects in children with cerebral palsy (CP).

**Subjects and Methods:**

A non-randomized controlled prospective study. Thirteen children with CP participated. A program of four weekly sessions lasting 65 minutes each was implemented over six weeks. Gross motor function was assessed using the 88 items Gross Motor Function Measure (GMFM-88); physical exercise endurance was measured with the Six-Minute Walk Test (6MWT) using the device; the number of steps walked in each session and mode of use was recorded to evaluate adaptation to the activity. Three evaluations were conducted: before treatment, at the end of treatment (6 weeks), and a follow-up evaluation at 12 weeks.

**Results:**

The total GMFM-88 score showed significant changes at the end of the intervention (*p* < 0.001), which persisted at follow-up (*p* < 0.001). The number of steps in automatic and active modes increased significantly after the intervention (*p* < 0.001) and were maintained at follow-up (*p* = 0.001). Lastly, the 6MWT improved significantly after the intervention, with a reduction observed at follow-up (*p* < 0.001).

**Conclusions:**

Six weeks of intensive training with ATLAS 2030 positively impacts the gross motor function of children with CP, with benefits increasing six weeks after treatment completion. Physical endurance and adaptation to the activity improve with continued use. These results support the potential of ATLAS 2030 as an intensive therapeutic strategy for this population.

## 1. Introducción y Objetivos

La Parálisis Cerebral (PC) agrupa a una serie de trastornos que son la causa de discapacidad neuromotora más frecuente en la infancia [1], afectando aproximadamente a entre 2 y 3 de cada 1000 nacimientos en países desarrollados [2, 3]. Las secuelas motoras de la PC afectan a la función motora gruesa y fina, así como a la coordinación y al equilibrio. Uno de los desafíos más notables que presentan estos pacientes es la rehabilitación de la marcha, que se ve comprometida por la espasticidad, la hipotonía o la disquinesia [4].

Los exoesqueletos pediátricos de marcha en superficie han surgido como una opción prometedora, al proporcionar soporte físico y facilitar la realización de movimientos controlados, lo que podría traducirse en mejoras significativas en la marcha [5, 6]. Toda la investigación disponible sobre exoesqueletos pediátricos de marcha en superficie es muy preliminar, y se ha hecho con pequeños grupos de pacientes con niños con atrofia muscular espinal (AME) Tipo II [7] y con niños con PC [8, 9]. En PC el rango de movimiento, la espasticidad y la fuerza mejoraron en una serie de casos [8].

El objetivo principal del presente trabajo es la valoración de los cambios en la función motora gruesa de niños con PC tras recibir un programa de entrenamiento de 6 semanas con el exoesqueleto ATLAS 2030 y tras 6 semanas después de la intervención. Los objetivos secundarios son medir los cambios en la adaptación y resistencia al ejercicio, así como el mantenimiento de los mismos tras la intervención.

## 2. Sujetos y Métodos

### 2.1 Diseño del Estudio

Estudio observacional de grupo único, llevado a cabo por el equipo de rehabilitación infantil del Hospital Aita Menni (País Vasco, España) en colaboración con la Asociación ASPACE Gipuzkoa y con el apoyo de la Diputación Foral de Gipuzkoa.

### 2.2 Participantes

Los participantes se reclutaron en la Asociación ASPACE Gipuzkoa, bajo los siguientes criterios de elegibilidad: diagnóstico de PC; 2–14 años de edad; nivel Gross Motor Function Classification System (GMFCS) III, IV o V.

Los criterios de exclusión para participar en el estudio fueron: espasticidad en los miembros inferiores mayor de 3 en la escala de Ashworth modificada, osteoporosis severa que se hubiera traducido en fracturas óseas sin trauma previo, y medidas antropométricas y rangos articulares que impidan el uso del exoesqueleto (peso del usuario ≥36 kg, ancho de caderas ≥35 cm, longitud del muslo fuera del rango 24–33 cm, flexo de rodilla ≤20º y flexión de cadera ≥100º).

### 2.3 Intervención

**Fig. 1.**
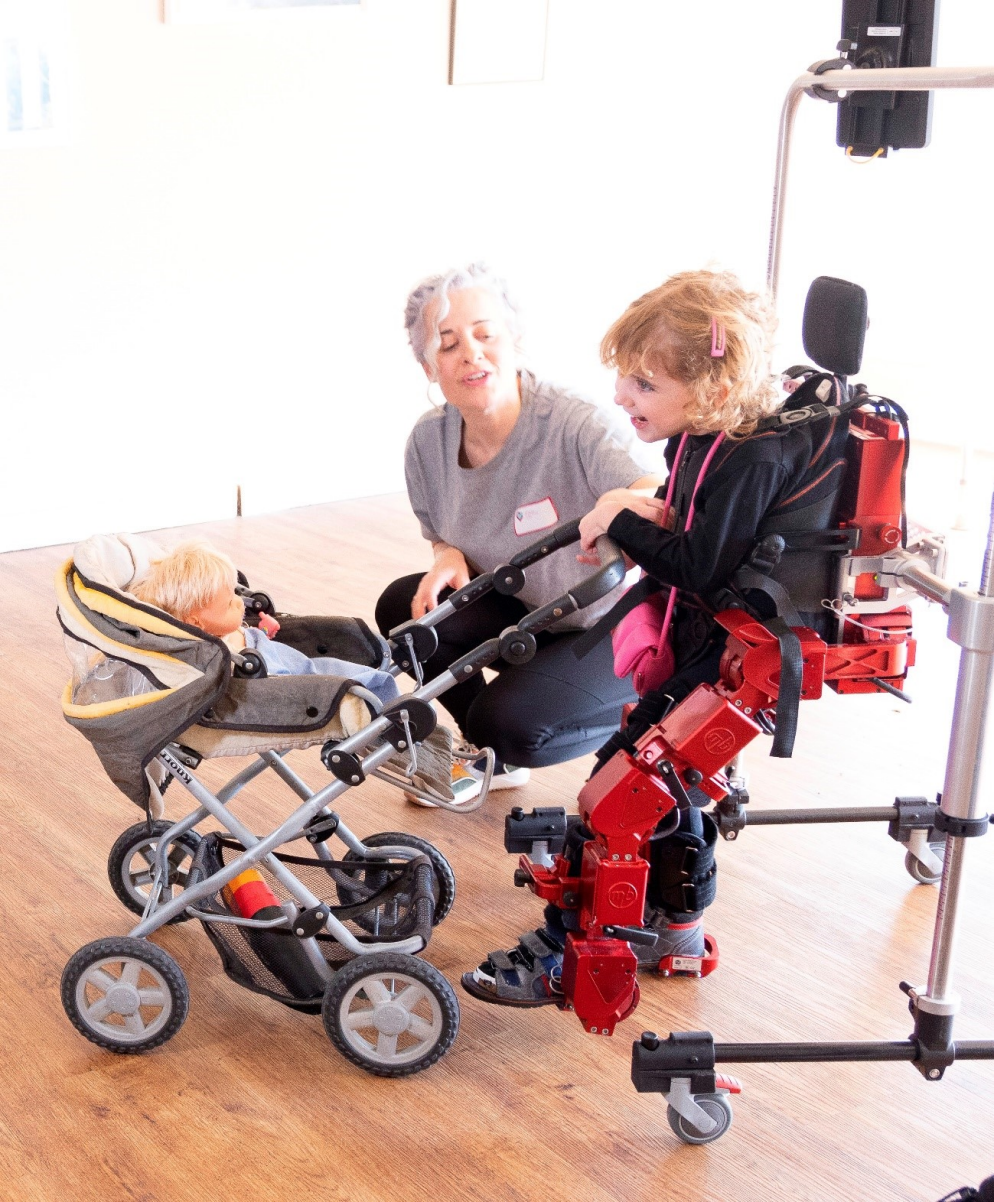
**Niña jugando y haciendo uso del exoesqueleto ATLAS 2030**.

La intervención consistió en 4 sesiones semanales de terapia con el dispositivo ATLAS 2030 durante 6 semanas. La sesión se protocolizó de la siguiente manera: colocación del exoesqueleto, 25 minutos marcha automática hacia delante, 15 minutos marcha en modo activo hacia delante, 15 minutos marcha automática hacia delante, 5 minutos marcha automática hacia atrás, 5 minutos marcha en modo activo hacia atrás, retirada del exoesqueleto Fig. 1. Durante la marcha se realizaron actividades basadas en objetivos terapéuticos según las capacidades de los participantes. Los niños que participaron en el estudio estaban escolarizados en escuela ordinaria o en centros de educación especial. Todos contaban con sesiones semanales de fisioterapia convencional que no se interrumpieron durante el estudio.

Fueron responsables de la intervención dos fisioterapeutas formadas en el uso del dispositivo y con experiencia en el campo de la neurorrehabilitación infantil, de 27 y 6 años respectivamente.

### 2.4 Dispositivo

El exoesqueleto ATLAS 2030 es un dispositivo portátil que proporciona asistencia de la marcha a través de ocho grados de libertad, cuatro por cada pierna, incluyendo rotaciones de cadera, rodilla y tobillo. Estudios preliminares han demostrado su seguridad y mejoras funcionales en pacientes con atrofia muscular espinal (AME) [10] y PC [8].

Permite caminar hacia adelante y hacia atrás, y ofrece dos modos de uso: (1) modo automático, asiste completamente la marcha del paciente siguiendo un patrón de referencia basado en la cinemática de sujetos sanos a la velocidad establecida, y (2) modo activo, en el que el movimiento se detiene durante la fase de oscilación hasta que el paciente supera un umbral de fuerza articular programado para continuar el movimiento. También permite movimientos desde sentado a de pie. Todas estas características son controladas desde una aplicación que se ejecuta en una tableta vinculada a la conexión Wi-Fi proporcionada por el exoesqueleto. Su marco de seguridad permite que el niño realice actividades con las manos y que el terapeuta trabaje delante del niño focalizándose en objetivos terapéuticos más allá de la seguridad y el posicionamiento, proporcionados por el exoesqueleto.

### 2.5 Medidas de Resultado

Las evaluaciones se realizaron en tres ocasiones: (1) antes de comenzar la intervención (V_0_); (2) al acabar la intervención a las 6 semanas (V_6_); y (3) en la valoración de seguimiento (V_12_) a las 6 semanas post-intervención con el objetivo de evaluar si perduraban los resultados.

Las medidas de resultado que se evaluaron fueron las siguientes:

1. Medida de resultado primaria:

- Gross Motor Function Measure de 88 ítems [11, 12] (GMFM-88): la escala contiene 88 ítems en una escala de 4 puntos (0–3) que se dividen en cinco dimensiones: (A) Decúbitos y volteos (17 ítems); (B) Sedestación (20 ítems); (C) Gateo y rodilla (14 ítems); (D) Bipedestación (13 ítems); y (E) Caminar, correr y saltar (24 ítems). Una mayor puntuación en la escala indica una mejor función motora gruesa y, por ende, una mayor consecución de hitos del desarrollo motor.

2. Medidas de resultados secundarias:

- 6-Minutes-Walk Test (6MWT): mide la distancia caminada en 6 minutos, con el objetivo de evaluar la resistencia física al ejercicio. En este caso, se realizó mientras se utilizaba el dispositivo en modo activo, hacia delante y umbral 1 [13].

- Pasos totales en modo activo hacia delante y hacia detrás, para medir la adaptación al ejercicio, en este caso, la marcha con el dispositivo.

No se realizaron otras pruebas de marcha al margen de las realizadas con el exoesqueleto ya que los niños carecían de capacidad de marcha autónoma (7 niños GMFCS V, y 6 niños GMFCS IV).

### 2.6 Análisis Estadístico

Las estadísticas descriptivas se utilizaron para resumir los datos cuantitativos, empleando la media y la desviación estándar (media ± desviación estándar). Para determinar el tipo de distribución de la muestra, se realizó una prueba de Shapiro-Wilk (n < 30) junto con gráficos de Q-Q e histogramas. En casos donde no se cumplían los supuestos de las estadísticas paramétricas, se utilizó una prueba t de muestras pareadas o una prueba de rangos con signo de Wilcoxon para evaluar las mediciones antes y después de la sesión. Se estableció un nivel de significancia de α = 0,05 y las diferencias se consideraron estadísticamente significativas cuando *p* < 0,05.

Se realizó un ANOVA de medidas repetidas de un sólo factor para comparar las medias de diferentes evaluaciones dentro del mismo grupo a lo largo del tiempo. El criterio de esfericidad se evaluó utilizando la prueba de Mauchly y se aplicaron medidas correctivas si los criterios no se cumplían. Se realizaron ajustes a los grados de libertad del numerador y del denominador multiplicando el factor de ajuste “ε” para las pruebas de efecto. La elección de epsilon se determinó mediante el epsilon de Greenhouse-Geisser, con un valor de 0,75. Si el valor excedía 0,75, se aplicaba el epsilon de Huynh-Feldt; de lo contrario, se usaba el epsilon de Greenhouse-Geisser. La presentación de los resultados del ANOVA cumplió con los estándares de la American Psychological Association (APA), incluyendo la estadística F, los grados de libertad, el nivel de significancia, el tamaño del efecto y la potencia estadística (1-β). Después de establecer las diferencias de medias, se realizaron pruebas de rango post hoc para identificar qué medias eran significativamente diferentes. Se empleó la prueba de Bonferroni, comparando las medias después de rechazar la hipótesis nula de medias iguales con la prueba ANOVA. Se reportaron la media, la desviación estándar y los intervalos de confianza del 95% de los pares de muestras con diferencias estadísticamente significativas (*p* < 0,05).

Todos los análisis y representaciones gráficas se realizaron utilizando RStudio® versión 2022.7.2.576 (RStudio, PBC, Boston, MA, USA), y Microsoft Excel® 2019 (Microsoft Corporation, Redmond, WA, USA).

## 3. Resultados

Participaron 13 niños con PC muy severa, todos ellos con GMFCS IV o V, con una edad promedio de 8,15 ± 2,88 años y un peso medio de 17,94 ± 5,85 kg. En la Tabla 1 se recoge la información de los participantes al inicio del estudio. No se registraron eventos adversos debidos al dispositivo. Sólo hubo una pérdida de un participante debido a una lesión física ajena al uso del exoesqueleto. El resto realizó todas las sesiones pautadas.

**Tabla 1.** **Información de los participantes al inicio del estudio**.

Participante	Edad	Sexo	Peso (kg)	GMFCS	GMFM-88
1	4	Femenino	13,2	IV	17,2
2	5	Masculino	11,5	IV	15,6
3	9	Masculino	22,3	IV	15,1
4	11	Femenino	13,7	V	11,4
5	12	Femenino	28,0	IV	32,4
6	11	Masculino	28,8	V	17,5
7	6	Masculino	14,0	V	13,1
8	10	Femenino	13,4	V	6,9
9	10	Masculino	19,2	V	24,7
10	11	Masculino	19,0	IV	32,8
11	5	Femenino	15,6	V	7,4
12	7	Femenino	22,0	V	12,9
13	5	Masculino	12,5	IV	28,5

GMFCS, Gross Motor Classification System; GMFM-88, Gross Motor Function Measure 88 items.

**Fig. 2.**
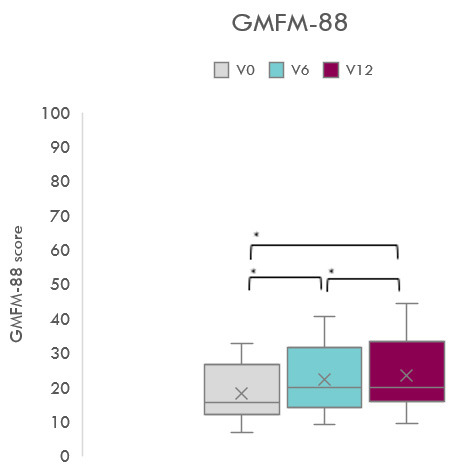
**Evolución de la función motora gruesa evaluada con la GMFM-88: Gross Motor Function Measure 88 items**. * *p* < 0,001.

La Fig. 2 muestra la evolución de la puntuación GMFM-88 a lo largo de las valoraciones. Se observaron diferencias estadísticamente significativas en las mediciones de GMFM-88 en las tres valoraciones, con un efecto de gran magnitud (F _1,92_ = 42,28; *p* < 0,001; η^2^ = 0,78; β-1 = 1). Las puntuaciones en la V_0_ (18,14 ± 2,44) fueron significativamente más bajas que en V_6_ (22,21 ± 2,87; *p* < 0,001 [IC 95% –5,63; –2,53]) y en V_12_ (23,56 ± 3,15; *p* < 0,001 [IC 95% –7,66; –3,193]). Además, las puntuaciones en V_6_ fueron inferiores en comparación con las puntuaciones en V_12_ (*p* = 0,022 [IC 95% –2,51; –0,18]). La Tabla 2 presenta las puntuaciones medias y los resultados de las pruebas estadísticas para cada una de las dimensiones de la GMFM-88. Los cambios significativos se concentran en las dimensiones “Decúbitos y volteo” y “Sedestación”. En la valoración de estas dos dimensiones se alcanzan valores significativos en las comparaciones entre V_0_ y V_6,_ y entre V_0_ y V_12_. No se observan cambios significativos entre V_6_ y V_12_. Las dimensiones de bipedestación y marcha presentan una mejora no significativa.

**Tabla 2.** **Evolución de las dimensiones de la GMFM-88 con pruebas estadísticas**.

D	F	*p*	η²	β-1	Eval	M ± DE	Co	IC	*p*
A	20,15	<0,001	0,63	1	V_0_	58,67 ± 16,53	V_12_ V_6_	–10,71; –3,46	<0,001
					V_6_	65,76 ± 17,62	V_12_V_12_	–5,74; 2,73	1
					V_12_	67,27 ± 18,09	V_12_ V_0_	4,43; 12,76	<0,001
B	30,31	<0,001	0,72	1	V_0_	24,10 ± 16,44	V_12_ V_6_	–11,11; –3,31	<0,001
					V_6_	31,41 ± 17,14	V_12_ V_12_	–4,66; 0,30	0,09
					V_12_	33,59 ± 18,24	V_12_ V_0_	5,34; 13,63	<0,001
C	3,96	0,06	0,25	0,5	V_0_	3,11 ± 5,36	V_12_ V_0_	–6,70; 1,57	0,3
					V_6_	5,68 ± 10,13	V_12_ V_12_	–2,31; 0,11	0,08
					V_12_	6,78 ± 11,16	V_12_ V_0_	–1,11; 8,44	0,16
D	2,90	0,11	0,19	0,4	V_0_	3,35 ± 5,38	V_12_ V_6_	–4,79; 0,52	0,13
					V_6_	5,49 ± 8,39	V_12_V_12_	–3,06; 1,81	1
					V_12_	6,11 ± 10,60	V_12_ V_0_	–1,76; 7,29	0,35
E	4,01	0,05	0,25	0,5	V_0_	2,35 ± 4,62	V_12_ V_6_	–2,06; 0,35	0,21
					V_6_	3,20 ± 5,62	V_12_V_12_	–2,27; 0,56	0,36
					V_12_	4,06 ± 6,81	V_12_ V_0_	–0,52; 3,94	0,16

D, dimensión; F, Fisher; *p*, *p* value; η^2^, Eta parcial cuadrado; β-1, potencia estadística; M, media; DE, desviación estándar; Co, comparación; IC, intervalo de confianza; V_6_, valoración post-tratamiento de las seis semanas; V_12_, valoración followup de las doce semanas; A, Decúbitos y volteo; B, Sedestación; C, Gateo y rodillas; D, Bipedestación; E, Caminar, correr y saltar.

**Fig. 3.**
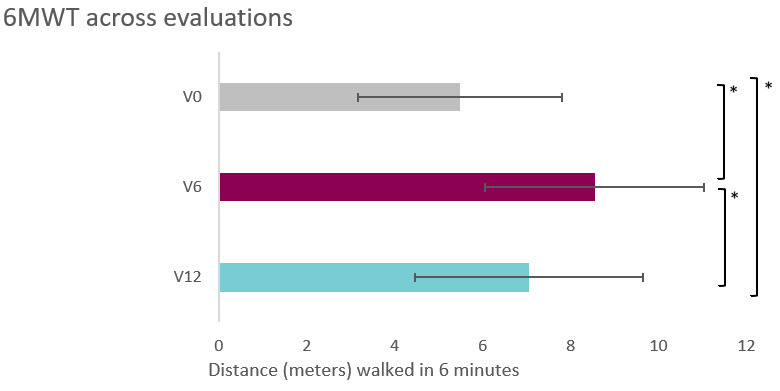
**6MWT across evaluations**. * *p* < 0,001. 6MWT, 6-Minutes-Walk Test.

Hubo diferencias significativas en la prueba 6MWT a lo largo de las sesiones (F _1,15_ = 29,13; *p* < 0,001; η^2^ = 0,71; β-1 = 1). La distancia recorrida en V_0_ (5,49 ± 0,72 metros) fue inferior a la de V_6_ (8,55 ± 0,70; *p* < 0,001 [IC 95% –4,57, –1,54]). La distancia recorrida en V_12_ fue inferior a la de la V_6_ (7,05 ± 0,6; *p* < 0,001; [IC 95% 0,69; 2,31]). En la Fig. 3, se refleja la progresión de la distancia recorrida durante la prueba del 6MWT a lo largo de las valoraciones.

**Fig. 4.**
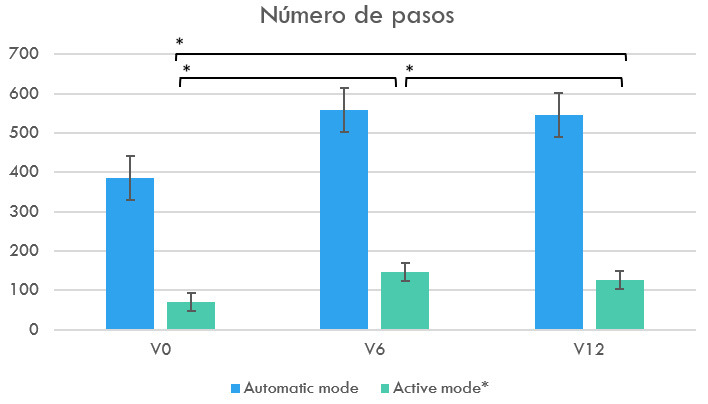
**Número de pasos en los tres momentos de evaluación**. * significa que hay una diferencia estadística significativa (*p* < 0,001) en el nḿ̆issingmero de pasos cuando se compara V0 con V6 o V12, y cuando se compara V6 con V12

El número de pasos en modo activo se incrementó en V_6_ con respecto a V_0_ (F_1,63_= 59,80; *p* < 0,001; η^2^ = 0,83; β-1 = 1). El número de pasos en modo activo en V_6_ fueron superiores que en V_12_ (*p* < 0,021 [IC 95% –37,00, –2,85]). La cantidad global de pasos en V_0_ (70,23 ± 9,02) fue significativamente menor que en V_6_ (145,85 ± 7,02, *p* < 0,001 [IC 95% –99,79; –51,44]) y V_12_ (125,92 ± 8,51; *p* < 0,001; [IC 95% –73,43; –37,95]). En la Fig. 4, se refleja la progresión en el número de pasos en cada modo de uso del dispositivo a lo largo de las valoraciones.

## 4. Discusión

Los resultados de este estudio indican que el exoesqueleto ATLAS 2030 tiene un efecto significativo en la mejora de la función motora gruesa en niños con PC niveles GMFCS IV y V; la mejoría alcanzada tras el uso del dispositivo sigue en aumento tras las 6 semanas de la intervención. Es notable que las dimensiones de la GMFM-88 que mostraron mayores beneficios están asociadas principalmente con los decúbitos/volteos y la sedestación (dimensiones A y B, respectivamente). Esto es particularmente relevante, ya que estas dimensiones están muy afectadas en la población estudiada y los resultados sugieren que entrenar la deambulación con ATLAS 2030 ha permitido mejorar dimensiones que son previas al desarrollo de la marcha en los niños. Pudiera argumentarse que los pilares iniciales de la marcha (enderezamiento, control postural, percepción del esquema corporal, disminución del empuje) vieran estimulado su desarrollo entrenando la marcha con el exoesqueleto. Los resultados en las dimensiones de bipedestación y marcha son de una mejora no significativa. Esta favorable evolución en la función motora gruesa está en línea con la literatura existente [8, 9]. Delgado* et al*. [8] mostraron mejoras en la movilidad, la fuerza muscular y la espasticidad en una pequeña serie de niños con PC. Estas variables no fueron el foco específico de nuestro estudio, pero son componentes clave que contribuyen a las mejoras en el control motor y postural observadas en nuestros participantes. De manera similar, Diot* et al*. [9], en un estudio de un caso con PC con GMFCS V, destacaron mejoras focales en la espasticidad de los flexores de rodilla y en el control postural, lo que refuerza la capacidad de los exoesqueletos robóticos para influir en la espasticidad y en el control motor.

Estos resultados suscitan la reflexión de si este tipo de entrenamiento propicia un cambio neurológico estable, o si es más correcto verlo como un aprendizaje motor. Es una pregunta difícil de contestar y depende en gran medida de lo que consideremos como “cambio neurológico”. Es indudable que los niños que mejoraron aprendieron a hacer cosas nuevas con ayuda del exoesqueleto, y eso se tradujo en unas mejores puntuaciones en el GMFM-88. Si este cambio refleja un progreso estable en la maduración motora, o simplemente un aprendizaje motor, es una cuestión conceptual y empírica importante, aunque quizás no muy relevante a nivel clínico, en donde el progreso funcional es central. También son importantes a nivel clínico los cambios en la calidad de vida, incluyendo aspectos como el fortalecimiento de la autoestima y el bienestar psicológico, tanto de los niños como de sus familias [14]. En este terreno las observaciones cualitativas realizadas en este estudio fueron elocuentes y merecen futura investigación empírica.

Otro hallazgo relevante es la mejoría en la resistencia física y en la adaptación al ejercicio, medido a través del 6MWT y del recuento de pasos en modo activo. La reducción observada en estas métricas seis semanas post-intervención subraya la necesidad de un entrenamiento continuado para mantener las mejoras en la resistencia física, especialmente en niños con una PC severa o muy severa.

La excelente aceptación del tratamiento por parte de los participantes y sus familias se refleja en la altísima adherencia al estudio; sólo un participante abandonó el estudio por una complicación somática sobrevenida e incompatible con las sesiones de tratamiento. Este aspecto es importante a la hora de generalizar cualquier estrategia de tratamiento. Estamos ante un estudio piloto y es todavía prematuro aventurar la frecuencia e intensidad óptima para el propósito rehabilitador. Es, sin duda, una tarea de investigación pendiente y prioritaria. Quienes trabajamos en la rehabilitación de niños con severas dificultades para la marcha contamos con una nueva herramienta de rehabilitación, y también con un producto de apoyo que permite, a los niños que no puedan llegar a andar, desplazarse en posición de bipedestación. Este modo de desplazamiento contribuye a la mejora en el funcionamiento fisiológico, en la interacción social y en la autoestima. El modo de incorporación del exoesqueleto a nuestros Servicios rehabilitadores y los análisis coste-efectividad son tareas de futuro. Lo que sabemos por experiencias previas es que la tecnología que aporta valor a la vida de las personas, se incorpora progresivamente y reduce sus costos a medida que se generaliza su producción y su demanda.

El estudio actual exhibe varias limitaciones que deben ser abordadas en futuras investigaciones: la ausencia de un grupo control, la falta de evaluadores ciegos y el reducido tamaño de la muestra limitan la fortaleza de las conclusiones. En futuras investigaciones sería deseable contar con medidas de resultado de la marcha complementarias a las ofrecidas por el uso del exoesqueleto. Al no existir un grupo control no puede descartarse que los cambios se deban a las otras terapias que reciben los niños, aunque ha de recordarse que los cambios en el GMFCS se produjeron en 6 semanas de entrenamiento con el exoesqueleto en un grupo de niños con problemas de marcha severos y crónicos.

## 5. Conclusiones

El entrenamiento intensivo con el exoesqueleto ATLAS 2030 mejora significativamente la función motora gruesa en niños con PC, y estimula su desarrollo psicomotor. Los objetivos secundarios también se cumplieron, observándose una mejora significativa de la adaptación y resistencia al ejercicio. Queda pendiente estudiar la dosificación y frecuencia óptima para maximizar la mejoría del funcionamiento motor.

## Data Availability

Las bases de datos utilizadas en este estudio están pueden ser solicitadas al autor responsable de la correspondencia.

## Contribuciones de los Autores

IG y NM aplicaron los tratamientos y recolectaron los datos, CL valoró la idoneidad de los niños para su inclusión en el estudio, PF se encargó de los análisis estadísticos, JM y JIQ diseñaron el estudio y participaron en la escritura del texto. Todos los autores contribuyeron a los cambios editoriales en el manuscrito. Todos los autores leyeron y aprobaron el manuscrito final. Todos los autores han participado lo suficiente en el trabajo y han acordado ser responsables de todos los aspectos del mismo.

## Aprobación Ética y Consentimiento Informado

El estudio se realizó siguiendo la Standard Protocol Items: Recommendations of Intervention Trials (SPIRIT) y el acuerdo de Helsinki. Se obtuvo la aprobación del Comité de Ética de la Investigación con Medicamentos de Euskadi (referencia PS2023001). Todas las familias de los niños fueron informadas debidamente y se obtuvo su consentimiento informado por escrito.

## Agradecimientos

A la Diputación de Gipuzkoa, a la Asociación ASPACE Gipuzkoa, a los niños y a sus familias que trasladaron diariamente a sus hijos y les acompañaron durante el estudio.

## Financiación

El estudio pudo llevarse a cabo gracias al apoyo financiero de la Diputación Foral de Gipuzkoa.
